# Patients’ needs and preferences in developing Art-Based Learning in outpatient palliative cancer care: A qualitative study

**DOI:** 10.1371/journal.pone.0342436

**Published:** 2026-02-17

**Authors:** Marike Geurts, Shailoh R. E. S. Phillips, Fabiola Camuti, Sabrina Kamstra, Jeroen Lutters, Famke Sinninghe Damsté, Silvia Russel, Gerben J. Westerhof, Michael Scherer-Rath, Hanneke W. M. van Laarhoven

**Affiliations:** 1 Department of Medical Oncology, Amsterdam UMC Location University of Amsterdam, Amsterdam, The Netherlands; 2 Cancer Treatment and Quality of Life, Cancer Center Amsterdam, Amsterdam, The Netherlands; 3 Department of Psychology, Health and Technology, University of Twente, Enschede, The Netherlands; 4 Department of Critical Creative Pedagogies, HKU University of the Arts Utrecht, Utrecht, The Netherlands; 5 Arts Department, Amsterdam UMC, Amsterdam, The Netherlands; 6 Department of Art Education as Critical Tactics, ArtEZ, Arnhem, The Netherlands; 7 Department of Empirical and Practical Religious Studies, Radboud University, Nijmegen, The Netherlands; Macau University of Science and Technology, MACAO

## Abstract

**Purpose:**

Art-Based Learning (ABL), an art pedagogical practice, may assist cancer patients by providing a meaningful experience through art viewing. However, little is known about what needs to be considered when developing an exhibition space for ABL in a palliative care setting. This study aimed at providing an overview of needs and preferences from a patient perspective.

**Methods:**

Patients were included through purposive sampling based on the following criteria: WHO performance status 0 or 1, ≥ 18 years, Dutch proficiency, and ability to come to the hospital. For the online sessions, access to the internet, a device, and a microphone were required. Patients participated in an ABL session either online or in the hospital followed by a semi-structured interview. The transcribed interviews were thematically analyzed using both deductive and inductive approaches.

**Results:**

Participants (n = 13) had a positive experience with ABL either online (n = 6) or in the hospital (n = 7). The results showed the multidimensionality of the patients’ needs and preferences regarding the exhibition and ABL, from preferences regarding the exhibition space and needs for accessibility both online and on-site, to personalization of ABL by the facilitator.

**Conclusion:**

Our study showed that the hospital and online museum are appropriate and accessible environments for an exhibition for ABL. A varied selection of artworks might contribute most to a meaningful experience. Furthermore, we identified the patients’ need for a person-centered approach in ABL in palliative care, in which not only the facilitator, but also health care professionals play an important role.

## Introduction

The study of art-based practices has become an important aspect of supportive care research for cancer patients receiving palliative care. A body of literature suggests that art-based methods can improve the quality of life of cancer patients in palliative care by reducing anxiety, depression, fatigue, pain, and nausea [1–8]. In addition, previous research showed the use of art-based practices in improving the quality of life by providing spiritual care [9,10]. Spiritual care is an important facet of palliative care, aiming at alleviating existential burdens instead of psychophysical ones [11]. Life events such as a cancer diagnosis might raise existential questions and uncertainties about one’s life [12]. Patients need existential support in dealing with this. Previous research has shown that narrative based methods could be useful for this patient population in dealing with existentially impact life events [13]. However, the evidence is scarce and more research is needed on this topic.

An example of such a method is Art-Based Learning [14]. Art-Based Learning is an art pedagogical practice, meaning that the goal is to learn from art rather than about art. It takes place in an exhibition space, such as a gallery or museum. the method constitutes of four steps of guided art-viewing as shown in Table 1: i) asking a personal question, ii) being chosen by an artwork, iii) discovering the possible worlds within the artwork, and iv) sharing the story [14]. Because of its narrative approach, we hypothesize that Art-Based Learning could support advanced cancer patients in processing existentially impactful life events. However, research on the practice of Art-Based Learning is limited and mostly focuses on educational contexts [14]. In a pilot study, Russel et al. (2023) showed that Art-Based Learning could facilitate meaning-making by providing a meaningful experience for cancer patients in outpatient palliative care, helping them make sense of their life after receiving a life changing diagnosis [15].

**Table 1:** Four Steps of Art-Based Learning [14].

**Step 1. Ask a personally relevant question**	The first step is to ask a personally relevant question, that is appropriate for you. It can be anything. After this, the participant chooses an artwork. Lutters describes this process as: “a process of daydreaming: the ultimate choice is largely determined by an unnoticed dialogue with (unconscious) desires, needs and fears.”(p. 18) [14]. It entails the ability to let oneself be appropriated or chosen by the artwork.
**Step 2. Listen to a speaking object**	In the second step the participant becomes the listener of the speaking object, or the artwork. This is done by a close reading of an artwork. It is a process of “receiving, registering and allowing transformation.”(p. 19) [14] (). In the Art-Based Learning session, this step translates to the participant writing down their observations as objective as possible, without any thoughts, feelings or interpretations.
**Step 3. Enter a possible world**	After familiarization with the artwork through a close reading, the participant engages in a self-experience by entering into a conversation with the artwork. The participant is invited to enter the artwork, the possible world, and write down their experiences in this world.
**Step 4. Tell one’s own story**	In the fourth and last step the participant is asked to form the experience of the possible world into a story, to assign meaning to it. This step leads to a new story, new knowledge, and ideally to an answer to the personal question stated in the first step.

Still, it is unclear what conditions we need to consider when organizing Art-Based Learning sessions in an outpatient palliative care context, especially when developing a space, and more specifically an art exhibition, that facilitates meaningful experiences. Previous research on developing art exhibitions or art viewing in palliative care has shown that choices made were informed by either art experts or caregivers [3,7,16]. Their methodology showed little collaboration between professionals and the targeted patient population in curating exhibition spaces for palliative care aims. However, patients’ voices are important, as they provide valuable insights on how to improve care that addresses their needs from their own perspective.

Therefore, this study aimed at articulating the patients’ needs and preferences to outline what needs to be considered when developing an exhibition space for Art-Based Learning for patients with incurable cancer. This study also examined if the method needs to be tailored for outpatient palliative care, as it is primarily used in educational settings. Considering the patient population’s frailty, we also aimed at improving the accessibility of Art-Based Learning by exploring both on-site and online exhibitions. This way, we provided an overview informed by patients’ preferences and needs, which can guide healthcare providers, art facilitators, and art professionals in curating an exhibition designed to conduct Art-Based Learning in outpatient palliative care.

## Methods

### Design

A qualitative research approach was adopted in which patients were asked to participate in a single Art-Based Learning session, either online or in the hospital, followed by a semi-structured interview on the exhibition and the method. In these interviews, the patients could speak from their experience with Art-Based Learning, and were thus seen as experts. This bottom-up approach aligns with the so called ‘curatorial turn’, shifting from top-down decisions by art professionals to a more collaborative, participatory approach [17]. The COREQ 32-item checklist was used to enhance transparency, comprehensiveness and reliability of this study [18]. The study was reviewed and deemed exempt from ethical approval by the Medical Ethical Committee of the Amsterdam Medical University. The committee determined that external ethical review was unnecessary, as the study did not fall under the Medical Research Involving Human Subjects Act (no. 2024.0068, February 7th 2024).

### Participants

Patients were included in the upper gastro-intestinal outpatient clinic of the department of Medical Oncology of the Amsterdam UMC. Purposive sampling ensured representation of the target demographic. The following inclusion criteria were used:

- ≥ 18 years
- Diagnosed with metastasized cancer
- WHO performance status 0 (asymptomatic) or 1 (symptomatic, fully ambulatory)
- Proficient in Dutch
- Able to independently come to the hospital
- *Or* have access to internet, a laptop or tablet, and microphone

Patients were first approached by their attending physician. The attending physician were instructed to only approach those patients without cognitive impairments. Patients who were interested in participating were provided with detailed information about the study by MG. To mitigate potential cultural barriers and ensure participants felt comfortable, MG emphasized that prior knowledge of or experience with art was not required, reinforcing the notion that they were the experts in their own experiences. This approach aimed to create a space for honest and open dialogue. After agreeing to participate, the patients received an information letter and a written informed consent form was obtained before the session. Inclusion continued until data saturation was reached, marked by a repetition in responses.

### An exhibition for Art-Based Learning

We organized an on-site and online Art-Based Learning exhibition using the Amsterdam UMC art collection. Fifty artworks were selected by SK and JL based on four concepts, which are described in Table 2.

**Table 2:** Four concepts for curating practice.

**1. Practicality**	Artworks already in the hospital as part of the collection presentation were used. How the artwork is presented, depends on practical aspects such as space, safety and light
**2. Empiricism**	The selection of the artwork is based on patient appreciation, on ‘what works’. This is informed by conversations with the health care workers and patients. This means that the selection is based on experience and observation instead of theory
**3. Demographic**	Patients of the hospital have very different backgrounds, which propels the development of collecting non-Western art. The choice of artwork at the different units is made with the demographic in mind. Artworks that represent the different cultures and backgrounds, but also artwork that reflects the city of Amsterdam, seem to work well. The same goes for artwork that is colorful and recognizable for the younger patients.
**4. Reversed narrative**	Instead of creating a narrative using multiple artworks, the artwork needs to be able to tell a story on its own. The patient or visitor doesn’t visit the hospital for the artwork. The artwork on its own needs to have something to offer, a story or a ‘possible world’, in the words of Lutters (2019) [14].

The artworks were arranged along three color-coded art routes in the hospital, as shown in Table 3, to examine the feasibility of an exhibition in a hospital environment, and test different types of exhibition spaces in terms of light, color, and amount of hospital visitors. Additionally, an online museum was created on framevr.io for the online sessions, featuring a 3D space that mimicked a traditional white cube and included the complete selection of 50 digitized artworks.

**Table 3:** Descriptions of art routes in the hospital.

**Yellow**	This route starts in a hallway, through the pediatric outpatient clinic to the atrium on the first and second floor of the outpatient clinic. The atrium provides lots of natural light. There is a lot of hustle because of the outpatient clinic. The sounds echo in the atrium.
**Blue**	This route starts in a health care unit and ends at the other side of the hallway at the administrative department. This route is compact, relatively dark and quiet.
**Red**	This route is between two commercial squares with shops and food in the hospital. Whereas the squares are quite noisy and busy, the hallway that connects them are more quiet, darker, but still are spacious enough to look at the artwork on the walls.

The sessions were led by professional art educators, trained and certified in guiding Art-Based Learning. FK facilitated the sessions in the hospital, while RK guided the sessions in the online museum. Both educators used the same protocol. However, based on RKs experience with the method online, we allocated more time for the introduction to explain the navigation tools, leaving less time per step. Each session was allotted 45 minutes.

### Data collection

The data were collected between April 2024 and July 2024. SP and FC designed an interview schedule presented in Table 4. The topics in the interview schedule were derived from the following preliminary and ongoing research: i) a literature review investigating factors that might contribute to a meaningful art-based method in palliative care (manuscript forthcoming); ii) data collected by SP from interviews on curatorial practice in palliative care with curatorial experts (manuscript forthcoming); and iii) the pilot study on Art-Based Learning [15]. The interview schedule for the online group was slightly modified to address the virtual space and location.

**Table 4:** Brief description of the interview topics.

Topic	Description
Impression of art-based learning	General impression of the Art-Based Learning session
Guidance: Outreach & facilitation	Questions regarding the outreach by the HCPs, and the facilitation and guidance provided by the Art-Based Learning facilitator
Selection of artworks	General impression of the exhibition, choice of artworks, and experience with art-viewing
Duration	Questions regarding duration of the session, and preferences regarding the frequency
Space & location: online & hospital	*Hospital*: impression and opinion of the hospital location, the hospital as an art space, and possible suggestions for improvement. In addition, the online museum was shown and the participants were asked their opinion regarding the online museum. *Online*: impression and opinion of the online museum and possible suggestions for improvement.
Setting	Questions regarding the one on one session, and thoughts and opinions regarding group Art-Based Learning
Participation	Questions regarding the possibility of participating in the development of an art exhibition for Art-Based Learning in a palliative care setting.

The interviews were conducted privately, either in a room or on Microsoft Teams, with only the patient and researcher present. Multiple interview strategies were used to explore cultural norms and encourage mutual understanding, such as reflexive listening, and direct or minimal encouragement [19]. The interviews were recorded and encrypted using the Philips Digital Pocket Memo device, and transferred using the Philips SpeechExec software. The audio files were transcribed verbatim, and deleted afterward. The transcriptions were anonymized. MG also took observational notes on the environment and choice of artwork during the art-based learning session. Demographic and clinical data were collected from the patients’ medical record.

### Data analysis

The demographic and clinical data were analyzed using SPSS (v28.0.1.1 (15)). MG conducted a thematic analysis of the qualitative data in MAXQDA 2022 [20]. The constant comparative method was adopted by starting the analysis after six interviews [20,21]. We chose to start with a deductive analysis. Since we were looking for specific information regarding the exhibition and Art-Based Learning, we first selected relevant transcript segments based on the interview schedule topics before coding [21]. Within these topic segments, MG conducted an inductive analysis, using in vivo coding to stay close to the patients’ experience [20]. After this, the in vivo codes were clustered based on subject, and recoded into higher level codes. This formed the basis of our preliminary coding schedule. Each new interview transcript was analyzed sequentially using this schedule. New codes were added, clustered and recoded. This process continued until preliminary themes could be identified [21]. By adopting the constant comparative method, we could determine data saturation after thirteen interviews. The preliminary themes were then refined by cross-referencing with the original text and the topic segments until the definition of overarching, conceptual rich themes, which are presented in the result section [20]. The results are supported by quotes that highlight the nuances of each theme. We also aimed to include as many participants’ voices as possible.

### Intersubjectivity

The interviews and analysis were performed by MG. The codes, grouping of the codes and assessment of the themes were regularly discussed with FC and HvL and reported by MG. The final definitions of the themes were reviewed by FC, HvL, MSR and GW in a team meeting in which suggestions were made and discussed until consensus was reached. Additionally, the findings were discussed with external researchers. Credibility was further enhanced through rapport building and ongoing contact with the participants. Member checking was omitted due to participants’ advanced cancer and vulnerability.

### Research team and reflexivity

#### Personal characteristics.

The interviews were conducted, transcribed and analyzed by MG (female), who has a background in art history (BA), cultural history (MA), and nursing (BSc). Due to her experience with palliative care patients, MG tended to focus the analysis on the patients instead of the exhibition. This was kept in balance by the interdisciplinary research team.

#### Relationship with participants.

The researcher had no prior relationships with the participants.

## Results

In total 21 patients were approached. Two patients did not meet the inclusion criteria (being able to come to the hospital independently, and have access to a laptop or tablet). One patient was not interested. Three patients missed the Art-Based Learning session due to forgetfulness or unknown reasons. Two patients dropped out of the online intervention after consenting, as they preferred to invest their time in something they felt would be more valuable. In the end, thirteen participants participated in an Art-Based Learning session and were interviewed (on-site: 7; online: 6). Repetition in responses indicated data saturation and the analysis showed consistent findings. On-site participants were evenly spread among the different art routes. The descriptive statistics of the demographic and clinical data are displayed in Table 5. The following paragraphs present the results from the thematic analysis

**Table 5:** Descriptive statistics of demographic, clinical and research data.

Variable	Total	On-site	Online
**Site % (n)**	100% (13)	53.8% (7)	46.2% (6)
**Age, years, mean (SD)**	66.8 (SD 9.8)	73.6 (SD 4.7)	58.5 (SD 8)
**Sex % (n)**			
**male**	61.5% (8)	71.4% (5)	50% (3)
**female**	38.5% (5)	28.6% (2)	50% (3)
**Primary cancer site % (n)**			
**esophageal**	23.1% (3)	42.9% (3)	0.0% (0)
**stomach**	61,5% (8)	57.1% (4)	66.7% (4)
**pancreas**	15.4% (2)	0.0% (0)	33.3% (2)
**Time since diagnosis metastasis, months, median (range)**	8 (2-119)	11 (8-119)	6 (2-20)
**Treatment % (n)**			
**1st line**	84.6% (11)	100% (7)	66.7% (4)
**2nd line**	15.4% (2)	0.0% (0)	33.3% (2)
**WHO status % (n)**			
**WHO 0**	53.8% (7)	57.1% (4)	50% (3)
**WHO 1**	46.2% (6)	42.9% (3)	50% (3)
**Museum experience % (n)**			
**Yes**	69.2% (9)	71.4% (5)	66.7% (4)
**No**	30.8% (4)	28.6% (2)	33.3% (2)
**Duration interview, minutes, mean (SD)**	31 (SD 6:45)	30 (SD 4)	32 (SD 9:30)
**Duration Art-Based Learning, minutes, median (range)**	65 (20-110)	72 (35-110)	51 (20-70)

### Impression of Art-Based Learning: enrichment

Even though some participants admitted initial skepticism, almost all of them reflected positively on their Art-Based Learning session. They described the experience as fun and inspiring by encountering something new and unexpected, such as new perspectives, insights or reflections about themselves or their situation. This could be understood as **enrichment**.

*“At the very beginning, I was really standing there thinking, what am I supposed to do with this? […] Anyway, I actually had it right from the first piece that I thought, oh yes, this is going to go deeper.” (P1)*

*“I think I’m going to look at myself in the mirror for a while and see if I can discover more about myself.” (Po8)*

*“This really symbolizes, I think unconsciously, what has been on my mind.” (Po2)*

### Outreach: empathy and understanding

Regarding outreach, **empathy and understanding** were identified as a theme, meaning that health care providers and researchers should be aware of the participants’ vulnerable situation. This was also illustrated by the fact that even though most participants were positive about the outreach via the hospital, some felt the health care providers’ timing was off or that inclusion during a medical appointment was inappropriate.

*“I say, [being ill] is a nightmare without sound. That’s where you are at, and then you are approached to participate.” (Po8)*

### Selection of artworks: variety

All participants expressed their appreciation for the selection of artworks, with **variety** being the key factor as illustrated by participants describing the different styles, media and genres represented. Additionally, even though some participants encountered artworks they did not connect with at all, in the end all participants were able to connect with an artwork, and experienced ‘being spoken to’ by their artwork of choice, which is characteristic of the Art-Based Learning method [14].

*“I think it’s important to have variety, to make sure that people can choose something they like or find interesting.” (Po3)*

### The hospital location: presentation, accessibility, and living environment

**Presentation** was first identified as a theme, meaning that the presentation of the artworks mattered. Participants preferred the spacious and dynamic red and yellow routes, while the blue route through office spaces was seen as chilly, uninviting, and grey. This tied in with participants appreciating the lively, public atmosphere of the hospital, leading to the theme **living environment,** the hospital as an extension of society. Lastly, **accessibility** was also highlighted, with familiar surroundings reducing participation barriers, alongside practical concerns like parking and travel time.

*“The surroundings keep changing. I found that very pleasant.” (P2)*

*“It is allowed in that place, where all kinds of things happen. Where people come with all kinds of emotions, who care about their own health, or their loved ones.” (P6)*

### The online museum: accessibility, use of technology and intangibility

For online participants, **accessibility** was an important theme too, as the online museum reduced physical effort and allowed participation from home. Furthermore, the **use of technology** was identified as a theme, discussed both positively and negatively. While it enabled remote access and preparation, some struggled with devices, mainly tablets, or navigating the platform. However, the facilitator’s quick adaptation to these issues was appreciated by those facing difficulties. Moreover, the online environment’s **intangibility** was an issue, as participants couldn’t gauge the scale of paintings, potentially reducing the experience’s impact. On-site participants also noted this after viewing the online museum.

*“This is so nice if you are physically not able to go anywhere.” (Po7)*

*“I already visited the museum beforehand.” (Po2)*

*“Because of the size, a painting can also be impressive […], now you are looking at a picture, all in the same size.” (Po6)*

### Facilitation: personalization, and facilitator collaboration

All participants spoke positively about the facilitators. The facilitators were able to tailor the Art-Based Learning to the participants’ needs with step-by-step guidance and flexibility, which can be summarized as **personalization**. Participants also valued the facilitators active participation in the session, as it fostered collaboration, support and overall enhanced their experience with Art-Based Learning. This led to the theme of **facilitator collaboration.**

*“I wasn’t finished after fifteen minutes […] I was very happy it wasn’t like, pens down, hora est.” (P5)*

*“The fact that [the facilitator] is doing the same exercise as you, only enhances the experience.” (P6)*

### Duration: feasibility

Most participants were satisfied with the duration of the session. **Feasibility** was identified as a theme. Table 4 shows that the median duration was 65 minutes, with outliers being 20 minutes online and 120 minutes on-site. While the set pace was experienced as positive, the facilitator also allowed participants to take more time if needed. This aligns with the theme of personalization.

*“That’s an hour, that’s fine. If that’s feasible, then that’s fine.” (Po7)*

### Setting: privacy and group collaboration

Although most patients were positive about the individual sessions, some participants reported different preferences toward this setting of Art-Based Learning. Two preferences were identified: **group collaboration** and **privacy**. The first group preferred to work in a group, sharing experiences and empowering each other while also possibly lowering barriers to participate. The latter group preferred one on one session because of the intimacy, safety and being able to focus on oneself.

*“Right now, I’m doing this for myself.” (Po7)*

*“In a small group, indeed […] I think with people in a similar situation. […] That is important to me, that way you can support each other in certain things.” (P3)*

### Participation: relevance and competence

Most participants were open to providing suggestions on the development of an exhibition, but not to co-creating. This related to **relevance,** illustrated by participants prioritizing other things in this phase of their life. Many also felt they lacked the necessary knowledge or skills, highlighting **competence** as key to participation.

*“I just don’t want any obligations at the moment besides what I already do, let’s say hobbies.” (Po6)*

*“That really is a specialism, in my opinion (P3).”*

## Discussion

In this study, we aimed at gaining insight in the patients’ needs and preferences that need to be considered when developing an on-site and online art exhibition for Art-Based Learning for patients with incurable cancer. In this section, we first discuss the findings regarding patients’ preferences and needs in an exhibition for Art-Based Learning followed by our considerations regarding tailoring of the method to best suit outpatient palliative care settings.

### An exhibition for Art-Based Learning: practical considerations and the importance of variety

Even though some participants had issues with the presentation of the artworks in the hospital, our results showed that the hospital is suitable for Art-Based Learning for multiple reasons. First, it was accessible because of its familiarity. Second, participants were appreciative of its living environment. These findings contradict a study by Lone et al. (2021), which created an in-house art gallery for inpatients to specifically provide a reflective space away from the hospital environment [7]. This contradiction may be due to the difference in patient populations, as outpatient participants may find it easier to distance themselves from the hospital environment than inpatients. Unfortunately, Lone et al. (2021) did not report on the reception of the gallery by the patient population [7].

If we focus on the online participants, we observed that they were particularly grateful for the possibility the online environment offered to participate in Art-Based Learning. Even though the use of technology posed some challenges for the participants, the facilitator was able to successfully address them. Additionally, the intangibility of the artworks could be an issue, although it is unclear whether this influenced the experience as a whole. Nevertheless, this suggests that accommodating an online experience could be a valuable addition to the on-site sessions for this particular patient population.

Interestingly, all participants were positive about the variety of artworks in the exhibition. Variety in the selection appeared to be an important consideration in other art-based methods as well [3,7,16]. Gore et al. (2022) showed that providing enough options for participants to choose from stimulated discussion on the patients’ situation mediated by the artwork [3]. The results suggest this could also be the case in our study. In addition, providing enough options could offer the participants opportunities to be spoken to by an artwork, to encounter something new or unexpected. Research has shown that such an encounter could open up new possibilities and new perspectives, possibly leading to transformation and consequently, meaning-making through narrative integration [22]. Further research is needed to investigate if, and how unexpected encounters in Art-Based Learning are related to such meaning-making processes.

### Art-Based Learning in palliative care: call for a person-centered approach

Our results suggested that only minor adjustments are needed to adapt Art-Based Learning for the palliative care setting. First, we found a divide in participants either preferring individual sessions or in a group. This divide challenges us to rethink the setting in which we offer Art-Based Learning. Furthermore, the estimated duration of 45 minutes seemed too short. The median duration of 65 minutes suggests that a duration of approximately 60 minutes is more suitable. This is also supported by the qualitative data and the results from the pilot study from 2023 [15].

What seemed important in these suggestions for change, is the adaptation to the individuals’ needs. According to Payne (2008), responding to the patients’ needs should be a core principle of art-based methods in palliative care [23]. We identified multiple patient preferences and needs that pointed towards the importance of a so-called person-centered approach. This means that the individuals’ values and preferences should inform the shared decision-making and guide the care provided [24]. According to the World Health Organization, person-centered care is considered a key aspect of palliative care [25]. Starting with outreach, the results showed the importance empathy and understanding. Personalization was an important theme in the guidance, meaning that the facilitators were able to tailor the Art-Based Learning to the participants’ needs. This was also the case regarding the duration of the intervention. Even though the aim was 45 minutes, the facilitator adjusted the length of Art-Based Learning to the participant’s pace. Furthermore, looking at accessibility, we showed that it is important to understand what the patient needs. The results showed this could be practical in nature by providing parking spaces, but also facilitating an online environment to accommodate patients suffering with fatigue or other treatment related symptoms. These themes can be understood as essential elements for person-centered care [24]. These findings suggests that the themes we identified could provide a good starting point to guide further development of Art-Based Learning for patients with incurable cancer in palliative care.

## Strengths and limitations

Several limitations in the study should be considered. First, the results are not generalizable due to the homogenous patient population, all with upper-GI cancer diagnoses, mostly male. Yet, this male perspective might be valuable to a field dominated by female participants, as seen in a recent systematic review on art therapy in cancer care, in which 87% of participants were female [26]. Second, the small sample size (n = 13) may limit the representability and validity of the results. However, data saturation was established, meaning that the sample was adequate and our findings consistent.

Last, the exhibition was designed based on assumptions and best practices due to the knowledge gap in developing art exhibitions for Art-Based Learning in palliative care. We tried to overcome this limitation by collaborating closely with an interdisciplinary team of art professionals and researchers from medical oncology, psychology, and art academies. This interdisciplinary approach, in which patient were positioned as experts, can be considered a strength of this study. Incorporating multiple perspectives can help us uncover blind spots in our research, as well as enrich our methodology and findings [27]. More importantly, including patients’ voices is crucial to informing, improving and tailoring art-based practices in palliative care, such as Art-Based Learning, to their specific needs and preferences.

## Conclusion

This study provided an overview, informed by the patient population, of patients’ preferences and values that can guide healthcare providers, art facilitators and art professionals in curating an exhibition aimed at delivering Art-Based Learning. By taking the patients’ needs and preferences into account, we have identified multidimensional aspects that should be considered in future decision-making regarding Art-Based Learning in palliative care. Based on our findings on patients’ experience with Art-Based Learning, future research is recommended to examine if, and how Art-Based Learning could facilitate meaningful experiences for patients with incurable cancer.

## Supporting information

S1 Dataset Demographic and clinical data of sample. (CSV)

S2 Checklist COREQ 32 item checklist. (DOCX)
