# Supplementary material for: Patients’ needs and preferences in developing Art-Based Learning in outpatient palliative cancer care: A qualitative study
Source: PLoS One. 2026 Feb 17;21(2):e0342436. doi: 10.1371/journal.pone.0342436 (PMC12912537; doi:10.1371/journal.pone.0342436)
Supplement: S2 Checklist — (DOCX) [file pone.0342436.s002.docx]

Patients’ Needs and Preferences in Developing Art-Based Learning in Outpatient Palliative Cancer Care: a Qualitative Study

| **Consolidated criteria for reporting qualitative studies (COREQ): 32-Item Checklist for Interviews and Focus Groups (Tong, Sainsbury, & Craig, 2007)** | | |
| --- | --- | --- |
| Item | Guide questions/description | Reported yes/no |
| **Domain 1. Research team and reflexivity** | | |
| Personal characteristics | | |
| 1. Interviewer/Facilitator | Which author/s conducted the interview or focus group? |  |
| 2. Credentials | What were the researcher’s credentials? |  |
| 3. Occupation | What was their occupation at the time of the study? |  |
| 4. Gender | Was the researcher male or female? |  |
| 5. Experience and training | What experience or training did the researcher have? |  |
| Relationship with participants | | |
| 6. Relationship established | Was a relationship established prior to study commencement? |  |
| 7. Participant knowledge of the interviewer | What did the participants know about the researcher? |  |
| 8. Interviewer characteristics | What characteristics were reported about the interviewer? |  |
| **Domain 2. Study Design** | | |
| Theoretical framework | | |
| 9. Methodological orientation and theory | What methodological orientation was stated to underpin the study? |  |
| Participant selection | | |
| 10. Sampling | How were participants selected? |  |
| 11. Method of approach | How were participants approached? |  |
| 12. Sample size | How many participants were in the study? |  |
| 13. non-participation | How many people refused to participate or dropped out? Reasons? |  |
| Setting | | |
| 14. Setting of the data collection | Where was the data collected? |  |
| 15. Presence of non-participants | Was anyone else present besides the participants and researchers? |  |
| 16. Description of sample | What are the important characteristics of the sample? |  |
| Data collection | | |
| 17. Interview guide | Were questions, prompts, guides provided by the authors? Was it pilot tested? |  |
| 18. Repeat interviews | Were repeat interviews carried out? If yes, how many? |  |
| 19. Audio/visual recordings | Did the research use audio or visual recording to collect data? |  |
| 20. Field notes | Were field notes made during and/or after the interview or focus groups? |  |
| 21. Duration | What was the duration of the interview or focus groups? |  |
| 22. Data saturation | Was data saturation discussed? |  |
| 23. Transcripts returned | Were transcripts returned to participants for comment and/or correction? |  |
| **Domain 3. Data analysis and findings** | | |
| Data analysis | | |
| 24. Number of data coders | How many data coders coded the data? |  |
| 25. Description of the coding tree | Did authors provide a description of the coding tree? |  |
| 26. Derivation of themes | Were themes identified in advance or derived from the data? |  |
| 27. Software | What software, if applicable, was used to manage the data? |  |
| 28. Participant checking | Did participants provide feedback feedback on the findings? |  |
| Reporting | | |
| 29. Quotations presented | Were participant quotations presented to illustrate the themes/findings? Was each quotation identified? |  |
| 30. Data and findings consistent | Was there consistency between the data presented and the findings? |  |
| 31. Clarity of major themes? | Were major themes clearly presented in the findings? |  |
| 32. Clarity of minor themes? | Is there a description of diverse cases or discussion of minor themes? |  |

**Reference:**

Tong, A., Sainsbury, P., & Craig, J. (2007). Consolidated criteria for reporting qualitative research (COREQ): a 32-item checklist for interviews and focus groups. *Int J Qual Health Care, 19*(6), 349-357. doi:10.1093/intqhc/mzm042
